# Low-Canopy Seagrass Beds Still Provide Important Coastal Protection Services

**DOI:** 10.1371/journal.pone.0062413

**Published:** 2013-05-28

**Authors:** Marjolijn J. A. Christianen, Jim van Belzen, Peter M. J. Herman, Marieke M. van Katwijk, Leon P. M. Lamers, Peter J. M. van Leent, Tjeerd J. Bouma

**Affiliations:** 1 Department of Environmental Science, Faculty of Science, Institute for Water and Wetland Research, Radboud University Nijmegen, Nijmegen, The Netherlands; 2 Spatial Ecology Department, Royal Netherlands Institute for Sea Research, Yerseke, The Netherlands; 3 Department of Aquatic Ecology and Environmental Biology, Faculty of Science, Institute for Water and Wetland Research, Radboud University Nijmegen, Nijmegen, The Netherlands; Swansea University, United Kingdom

## Abstract

One of the most frequently quoted ecosystem services of seagrass meadows is their value for coastal protection. Many studies emphasize the role of above-ground shoots in attenuating waves, enhancing sedimentation and preventing erosion. This raises the question if short-leaved, low density (grazed) seagrass meadows with most of their biomass in belowground tissues can also stabilize sediments. We examined this by combining manipulative field experiments and wave measurements along a typical tropical reef flat where green turtles intensively graze upon the seagrass canopy. We experimentally manipulated wave energy and grazing intensity along a transect perpendicular to the beach, and compared sediment bed level change between vegetated and experimentally created bare plots at three distances from the beach. Our experiments showed that *i*) even the short-leaved, low-biomass and heavily-grazed seagrass vegetation reduced wave-induced sediment erosion up to threefold, and *ii*) that erosion was a function of location along the vegetated reef flat. Where other studies stress the importance of the seagrass canopy for shoreline protection, our study on open, low-biomass and heavily grazed seagrass beds strongly suggests that belowground biomass also has a major effect on the immobilization of sediment. These results imply that, compared to shallow unvegetated nearshore reef flats, the presence of a short, low-biomass seagrass meadow maintains a higher bed level, attenuating waves before reaching the beach and hence lowering beach erosion rates. We propose that the sole use of aboveground biomass as a proxy for valuing coastal protection services should be reconsidered.

## Introduction

Biological structures located in coastal sub- and intertidal ecosystems can attenuate waves and as a result directly contribute to coastal protection [1]–[3]. Both reef forming taxa such as corals [4], mussels [5] and oysters [6] and macroalgae and macrophytes such as kelp [7], seagrass [8], mangrove [9] and salt-marsh vegetation [10]–[12], are well known for their capacity to attenuate waves (see [1] for a review). As a consequence of the reduction of hydrodynamic energy, macrophyte vegetation typically accumulates sediment causing the water above the fore- or nearshore to become shallower [14], [15] (but see [16], [17]). Such sediment accretion also contributes to coastal protection, because wave attenuation increases with decreasing relative water depth [18]. The bathymetric wave-attenuating effect of vegetation-induced sediment accretion becomes especially important for those vegetation types that have a relatively small direct wave attenuating effect via their aboveground biomass. This applies for example to meadows of relatively short and highly flexible seagrass plants, which have limited wave-attenuating capacity by their canopy compared to stiffer vegetation [12]. If the structural complexity of such short vegetation is degraded further, *e.g*. due to a high grazing intensity, it becomes unclear to which extent they can still contribute to coastal protection.

Although sediment stabilization is often acknowledged as an important ecosystem service of seagrasses [19], [20] and anecdotic evidence points at increased erosion after a seagrass meadow has been lost (*e.g*. [13], [21]), experimental evidence for the exact mechanisms involved in sediment stabilization remains scarce. Seagrass meadows have been shown to attenuate hydrodynamic energy from currents [22], [23] and waves [12], [24], [25] and thereby trap suspended sediment and cause sediment accretion [14], [26]–[30]. However, with respect to sediment stabilization, most studies only refer to the effect of the canopy in the reduction of the hydrodynamic forces that may reach the sediment and impose a bed shear stress (τ_b_) to the sediment [31]. It has been suggested that belowground biomass of rhizomes and roots can stabilize sediments by altering the erodability as the critical bed shear stress (τ_crit_) is increased [31]. However, the relative importance of this mechanism is generally hard to study without disturbing the seagrass meadow and is, therefore, generally not addressed when studying the role of these macrophytes for coastal protection.

In the tropics, seagrass meadows typically occur on shallow reef flats in subtidal nearshore areas. In general, seagrass growth is often controlled by temperature, light availability and freshwater input but also by physical disturbance from waves and associated sediment movement [32], [33]. Top-down effects can also drive seagrass growth by the foraging of large herbivores. Recent studies in Bermuda, India and Indonesia reported intense grazing of green turtles on seagrasses [34]–[36], with harvesting rates up to 100% of the daily leaf production [36]. As a consequence, these heavily grazed meadows can have an extremely sparse cover with a low aboveground biomass (±10 g DW m^−2^) and short (<5 cm) canopy, while maintaining a high belowground biomass (±50 g DW m^−2^) [36]. This is in strong contrast to ungrazed meadows, where the aboveground biomass can be at least 10 times higher (*e.g*. biomass 118 g DW m^−2^, canopy height ±25 cm, described in [37]. Such grazing-induced alteration of the canopy structure makes these meadows interesting models to study the contribution of belowground tissues to coastal defense.

In this study, we therefore question *i)* if intensively grazed seagrass meadows with a very low-biomass canopy contribute to coastal protection by stabilizing the sediment against wave induced erosion, *ii)* if the importance of the sediment stabilizing effect of seagrass changes along a cross-shore profile and *iii)* if the sediment stabilization by seagrass meadows depends on the height of the canopy. To answer these questions, we experimentally manipulated seagrass above- and below-ground cover, wave forcing and grazing intensity along a transect between the reef and the beach.

## Methods

### Field site

**Figure 1:**
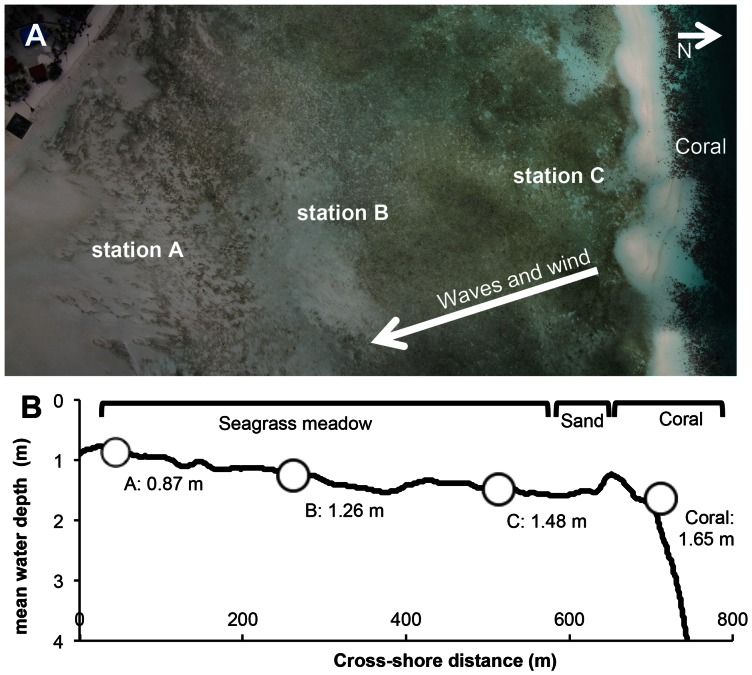
Location and depth-profile of the experimental site. (**A**) Aerial photo of the field site showing the locations of the stations, the seagrass bed on the reef flat in the subtidal nearshore area (light blue), and the coral drop off (transition to dark blue). See [36] for a more elaborate map. Waves are coming predominantly from the north (right). (**B**) Depth profile at increasing distance from the beach. Location of stations are indicated including their mean water depths.

The study was conducted on a subtidal seagrass meadow that covers the fringing reef flat of Derawan Island (Fig. 1a), Indonesia (2°17'19'N, 118°14'53'E; see [36] for a map and more details). The seagrass meadows are dominated by *Halodule uninervis* (Ehrenberg, Ascherson) growing on carbonate sediment. The carbonate substrate had a median grain size of 591±30 μm (*d*_50_, mean ± SE, Malvern Laser Particle Sizer) and did not differ significantly between stations. The canopy was of low structural complexity as a result of intensive grazing by green sea turtles (*Chelonia mydas,* 20.6 individuals ±2.2 ha^−1^, [38]). The hair-like leaves were short (<5 cm), narrow (<1 mm) and thin (<0.2 mm). Shoot density was 3335±224 shoots m^−2^ and shoots only had 1.8±0.1 leaves per shoot [36]. Aboveground biomass was 11.4±0.7 g DW m^−2^, and belowground biomass 52.0±4.5 g DW m^−2^. During the experiment (December 2011 –February 2012) spring tidal range was 2.9 m.

### Survey of reef flat depth profile

We mapped a cross-shore depth profile during slack low tide from the beach starting at the low water line, over the reef flat, to the coral reef. Water depth along the profile was measured by dragging a pressure logger (Sensus ultra, Reefnet Inc., Ontario, Canada) over the seabed at a fixed speed and simultaneous logging of time and position using a hand held GPS (GPSMAP 60CSx, Garmin, Olathe, USA). We averaged depth readings (obtained with a frequency of 1 Hz), using a sliding window over a 60 second interval, to reduce noise as a result of water level fluctuations caused by waves and methodological errors. Finally, the cross-shore depth profile is recalculated, based on the average burst reading by the wave gauges (see below ‘Wave measurements’), to get the average water depth over time.

### Wave measurements

We measured hydrodynamic forcing along the reef flat as a result of waves at four stations along the depth profile given in Fig. 1, using self-logging pressure sensors (Wave gauge: OSSI-10-003C, Ocean Sensor Systems, Coral Springs, USA). The instruments were placed at a height of 0.1 m above the bed. Three pressure sensors were placed in the seagrass meadow on the reef flat, and one sensor measured the waves coming in from the ocean over the reef crest at increasing distance from the shore (stations ‘A’ 45 m, ‘B’ 262 m, ‘C’ 513 m,‘Coral’ 712 m from the shore; see Fig. 1b). Wave heights were measured under a range of offshore wave conditions and tidal elevations during the whole experiment. A total of 3140 recording bursts were collected at a sampling rate of 10 Hz for 4 minutes, every 20-minutes. Recordings comprise a total of 209 hours of wave measurements (over a 44 day period, during rainy season). During the deployment of the wave gauges we caught a storm event (January 27, 2012, bursts 2879 to 3074), with peak wind speeds reaching 19 m s^−1^ from the north – northwest (±335°). We calculated wave attenuation values relative to the waves coming in at the reef station (Coral) for each station at the vegetated reef flat.

The obtained high frequency wave records were processed according to the following sequence: *(1)* pressure readings were converted to water level fluctuations (*η*), *(2)* erroneous spikes, shifts and corrupted bursts were removed from the data, *(3)* low-frequency tidal components were removed from each burst by detrending the water level fluctuations using a polynomial fit *(4)* from the detrended data significant wave heights (*H_s_*) (*cf*. *e.g*. [10], [39]) were calculated (c.f. [40]): 
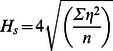
(1)in which *n* is the number of water level records in each burst (*n = *2400). In addition, we corrected the calculated significant wave height for the attenuation of the wave pressure field with depth and wave period [39]. From the detrended data, peak wave periods (*T_z_*) were computed based on zero-upcrossings [41].
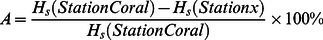
(2)


### Bottom shear stress calculation (τb)

Because the influence of waves on the sediment bed strongly depends on water depth, we calculated the wave-related bottom shear stress (*τ_b_*) over time (*c.f.* [42], [43]):
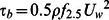
(3)where ρ is the water density, *f2.5* is the grain roughness friction factor calculated as 2.5d50. The wave height related orbital velocity at the bed (U*w*) was estimated using [44]:
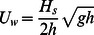
(4)in which g is the gravitational acceleration, and h is the mean water depth.

### Experiments to test and clarify sediment stabilization by seagrass

To test effects of seagrass presence on sediment stabilization, we compared the changes in sediment level inside bare sediment gaps (*i.e.*, 60×30 cm) to those inside a grazed seagrass meadow at T_0_ (*n = *5). These measurements were repeated at 3 stations, station A, B and C (for description see section “wave measurements”) to test for possible effects of different hydrodynamic forcing along the reef flat. Gaps were created at day 1 of the experiment by cutting the roots and rhizomes around a frame (60×30 cm) and removing all plant biomass within this frame. The size of the gaps was scaled to plant size and to dimensions of turtle gaps.

**Figure 2:**
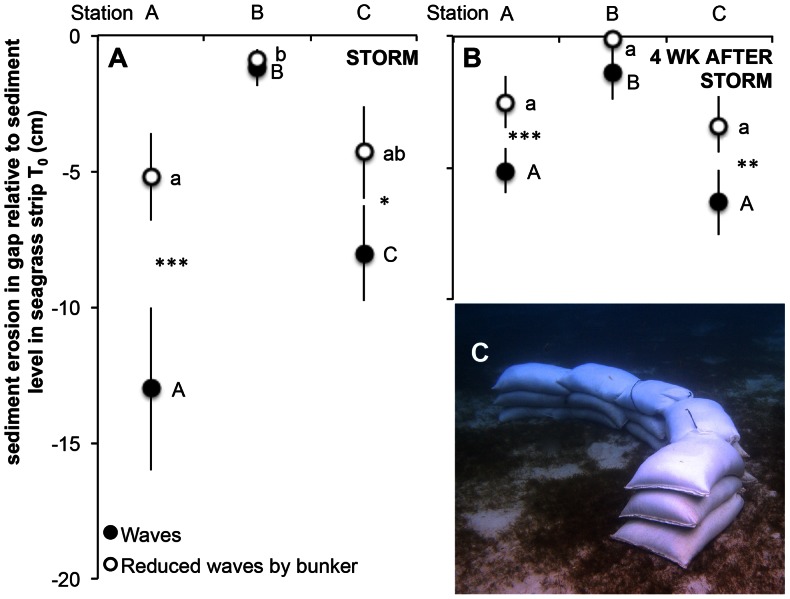
The effect of seagrass presence on sediment stabilization. Sediment levels in unvegetated gaps compared to levels in the seagrass meadow at T_0_ for two treatments: gaps exposed to waves (black circles) or exposed to waves reduced by wave bunkers (white circles). Seagrass stabilizes sediment both (**A**) directly after a storm and (**B**) 4 weeks after a storm. The inlay shows the setup of a bunker to reduce wave energy to seagrass and unvegetated gaps behind (left of) the bunkers. Significant differences between stations are indicated by different letters, and between wave exposed and wave-reduced plots by stars.

To test if waves were driving the erosion, we manipulated wave action entering the plots. Wave reduction was achieved by constructing bunkers of sandbags (50 kg) which were piled up (W:H; 5:3 bags; ±3:0.75 m) in a semi-circular shape to protect plots that were situated 30 cm behind the bunker (Fig. 2c). We compared plots with and without this wave reduction treatment, by measuring 5 replicate plots at three stations (*n = *15, in total).

**Figure 3:**
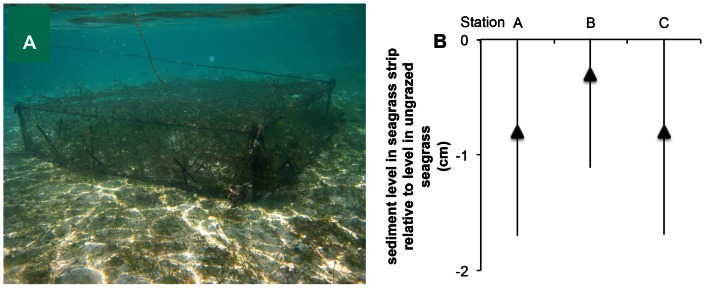
Effect of canopy length on sediment stabilization. (A) Turtle exclosure. (B) Difference in sediment bed level between grazed and ungrazed seagrass strips for the three stations (A, B, C) after 2 months protection by the turtle exclosure. The difference in leaf length of the canopy in turtle exclosures was a factor 2.6 longer (117.8±16.6 mm) than in grazed meadows (45.8±11.6 mm).

To study the effect of canopy leaf surface area on sediment stabilization we compared sediment bed level in grazed plots with ungrazed plots that were protected from turtle grazing for 2 months (Fig. 3a). Measurements for both treatments were replicated 5 times for each station. Plots comprised seagrass strips (60×15 cm) bordered by 2 bare sediment gaps (60×30 cm). We used exclosures (1.2×1.2×0.3 m, 5 cm mesh, Fig. 3a) that were designed to maximize light passage and minimize wave attenuation while excluding grazing of green turtles. Exclosures consisted of fishing net attached to the tops of four steel poles that were connected to ropes [36], and were inspected and cleaned trice a week. Wave attenuation by exclosures was minimal as weight loss of plaster sticks placed in and outside cages exposed to waves did not differ significantly. The seagrass canopy height was determined by measuring lengths of 28 shoots from cores (Ø 23 cm) in grazed (*n = *35) and ungrazed seagrass plots (*n = *15).

The experimental plots were selected at a location with homogeneous seagrass substrate, with minimum distances of 15 m between them. The plots of each station were located in a zone with minimal differences in water depth (20 cm) and were placed at a line parallel to the shore. Treatments were randomly assigned to the plots.

### Evaluation of sediment change

Quantitative measurements of changes in bed level were obtained using a sediment elevation bar method (SEB, *e.g*. [45], [46]) at the start and the end of the experiment. A long metal pin (150 cm) was inserted into the sediment as a reference at the start of the experiment. A horizontal bar of 150 cm, attached to a second vertical pin was placed on top of the vertical reference pin at each measurement until the horizontal bar touched the reference pin and was level. The distance between the horizontal bar and the bed surface was measured at 9 points, at a diagonal line over each experimental plot, during each measurement. The relative erosion during the experiment was determined as the difference between T_0_ and T_end_ values. This method was estimated to have an accuracy of 5 mm.

During the experiments the sediment erosion in the gaps was also scored visually in a semi-quantitative way (unchanged: ‘−’, minimal erosion: ‘±’, medium erosion: ‘+’, strong erosion ‘++’). These estimates were performed every 3^rd^ day during maintenance checks of all experimental plots, and data were converted to sediment erosion rates using a conversion factor that we derived from plots with both quantitative and semi-quantitative measurements for the same day.

### Evaluation of the wave reduction treatment

To evaluate the wave reducing effect of the sandbag bunkers, without having more wave loggers available, we compared weight loss of plaster sticks deployed inside and outside a bunker, at 3 locations along the reef flat. Relative weight loss by dissolution of the plaster is considered a proxy for hydrodynamic forcing and integrates effects from tidal currents and waves [47], [48]. Sticks were placed at seagrass canopy level at the seagrass – gap border (*n = *5 for each seagrass station) on a day with a large tidal difference, with sticks staying submerged continuously. Plaster sticks were molded using 20 ml of model plaster attached to the plungers of 60 ml syringes of which tips had been cut off. The sticks were weighted before and after 24 hours of placement at the plots, after drying until constant weight.

### Statistical analyses

A one-way ANOVA was used to analyze differences in wave height between stations. Two way ANOVA's were used to analyze the effect of station and wave reduction on sediment erosion and current velocity, and to analyze the effect of canopy length on sediment bed level. Data were log-transformed when necessary to meet assumptions for the ANOVAs. To evaluate possible differences between stations, we used Tukey HSD post hoc tests and for all hydrodynamic parameters we used Dunnett's post hoc tests for which we report *P*-values. Differences at *P<*0.05 were considered significant. R (version 2.15.1, June 2012) was used for all analyses. Results are presented as means ± their standard errors, unless stated otherwise.

## Results

### Hydrodynamic forcing

Mean significant wave heights (*H_s_*) differed significantly between stations along the reef flat, except for stations A and C (Table 1). During normal conditions (periods without storms), significant wave height from waves coming in from the sea onto the reef (at station Coral) was on average 0.19 m with an average peak period of 6.06 s (Table 1). During the storm in January, incoming significant wave height increased to an average of 0.40 m, with a peak value of 0.78 m (incoming waves at the station Coral, see Table 1). Typically, wave height decreased from the coral, over the vegetated reef flat, towards the beach as is shown by the lower average significant wave heights at stations C to A and the average relative wave attenuation (% in Table 1). Because there was very little standing canopy biomass to attenuate wave energy, this must be mainly the consequence of the decreasing water depth (Fig. 1b). However, at certain configurations of wave height and water depth, wave height started to increase, which is a typical consequence of shoaling or wave breaking. This increase in wave height was observed at all three stations on the reef flat (stations A, B and C Table 1; shoaling is wave attenuation <0), but at the station nearest to the beach (A) it occurred most frequently. Here, significant wave height could increase up to 1.8 fold (wave attenuation of -88.2% in Table 1) relative to the incoming wave height. Such increase is most probably due to wave breaking.

**Table 1:** Summary of the measured significant wave height (H_s_), peak wave period (T_z_) and bed shear stress (BSS) along a cross-shore seagrass profile (Fig. 1).

Station	Hs Mean		Hs Maximum		T_z_	Wave attenuation (normal)			BSS Mean		BSS Maximum	
	normal (m)	storm (m)	normal (m)	storm (m)	(s)	min	normal	max	normal (Pa)	storm (Pa)	normal (Pa)	storm (Pa)
A	0.15^a^ ±0.09	0.23^as^ ±0.19	0.52	0.68	5.13^c^±1.96	-88%	18%	100%	0.046^a±^0.034	0.111^abs±^0.101	0.22	0.40
B	0.13^b^±0.07	0.24^abs^ ±0.17	0.45	0.72	5.27^b^±1.84	–45%	30%	100%	0.026^b±^0.020	0.083^bs±^0.077	0.14	0.36
C	0.16^a^±0.07	0.30^cs^ ±0.17	0.48	0.74	5.27^b^±1.53	–28%	11%	100%	0.034^c±^0.026	0.113^bs±^0.086	0.19	0.38
Coral	0.19^c^±0.07	0.40^ds^±0.12	0.49	0.78	6.06^ a^±1.41				0.044^a±^0.034	0.207^cs±^0.115	0.23	0.66

Means with their standard deviations and maximum significant wave heights are given for normal conditions (n = 2945, “normal”  =  periods without storms) and during the storm (n = 195). Wave attenuation values less than 0 indicate wave shoaling.

The impact of waves on the reef-flat bed, estimated as the bottom shear stress (BSS), showed roughly the same trend as the significant wave heights. That is, BSS differed significantly between stations (*P<*0.05, Table 1). The relative wave height (the significant wave height relative to the water depth, *H_s_/h*) at station A was exceptionally high compared to the other stations, which means that the wave height was not yet accommodated to the local water depth. As a consequence, wave friction with the seabed might cause wave breaking, resulting in high turbulence and (swash and rip) currents at station A.

The wave bunker treatment (Fig. 2c) was effective in that it significantly reduced weight loss from the plaster sticks, indicati0ng that hydrodynamic energy was significantly lower behind the sandbags compared to plots fully exposed to waves (*P = *0.01).

### Sediment stabilization

Seagrasses significantly reduced sediment erosion by waves, although the degree of the erosion reduction strongly depended on the location along the reef flat (Fig. 2a and b). After a period of 2 months, stations A and C showed significant erosive bed level change in artificially created bare plots (*P<*0.01, Fig. 2b). At station B the sediment was not significantly eroded, which is in line with the lower hydrodynamic forcing measured at this station (Table 1). After a storm event, the sediment erosion was higher (Fig. 2a). The effect of waves on sediment erosion was largest at the nearshore, ‘swash’, zone around station A and close to the reef crest, ‘breaker’, zone around station C. This was demonstrated by the markedly lower sediment bed level at station A, than that at station B (*P = *0.02) and station C (*P<*0.001)(Fig. 2b). When exposed to waves, sediment level in the unvegetated gaps was eroded with, on average, 5.1 cm at station A, 6.3 cm at station C and only 1.3 cm at station B in 66 days (Fig. 2b). Right after the storm event, the sediment erosion in wave exposed plots at station A was a factor 2.5 higher (−13.0 vs. −5.1 cm, *P<*0.001) compared to erosion four weeks after the storm (Fig. 2a and b), but erosion was not significantly higher for station B and C after the storm.

Interestingly, the turtle exclosures revealed that grazed and ungrazed seagrass vegetation stabilize the sediment equally well. That is, excluding grazing did not cause any difference in sediment bed level compared to the grazed treatment (Fig. 3b), even though leaf length of the canopy in grazing exclosures was a factor 2.6 longer (117.8±16.6 mm) than in grazed meadows (45.8±11.6 mm).

The wave bunker treatment was effective in that it significantly reduced weight loss from the plaster balls, indicating that hydrodynamic energy was lower behind the sandbags compared to plots fully exposed to waves (*P* = 0.01,).

## Discussion

Coastal protection and sediment stabilization by seagrass is often valued as an important ecosystem service, which generally has been attributed to seagrass canopy properties [12], [24], [25], [29], [30]. This raises the question to which extent seagrass meadows that have very little canopy and have most of their biomass in belowground tissue can still contribute to coastal defense by stabilizing sediments. Present results convincingly demonstrate that even intensively grazed subtidal seagrass meadows, with a very short canopy, can still stabilize sediments effectively. This effect could be due to the remainder of the canopy, but although the seagrass has a relatively high density (±3000 shoots m^−2^), the leaves are extremely short and narrow. The aboveground biomass is minimal (±10 g m^−2^) and the percentage cover of the sediment is very low (<25 %). It is much more likely, therefore, that the difference in erosion between grazed vegetation and bare soil under high wave conditions is due to the role played by the relatively high belowground biomass. Roots and rhizomes can stabilize the sediment by reducing its erodability. This is an important novel addition to the findings of previous studies, which identified the hydrodynamic effect of the canopy as the only essential mechanism in sediment stabilization [12], [22].

**Figure 4:**
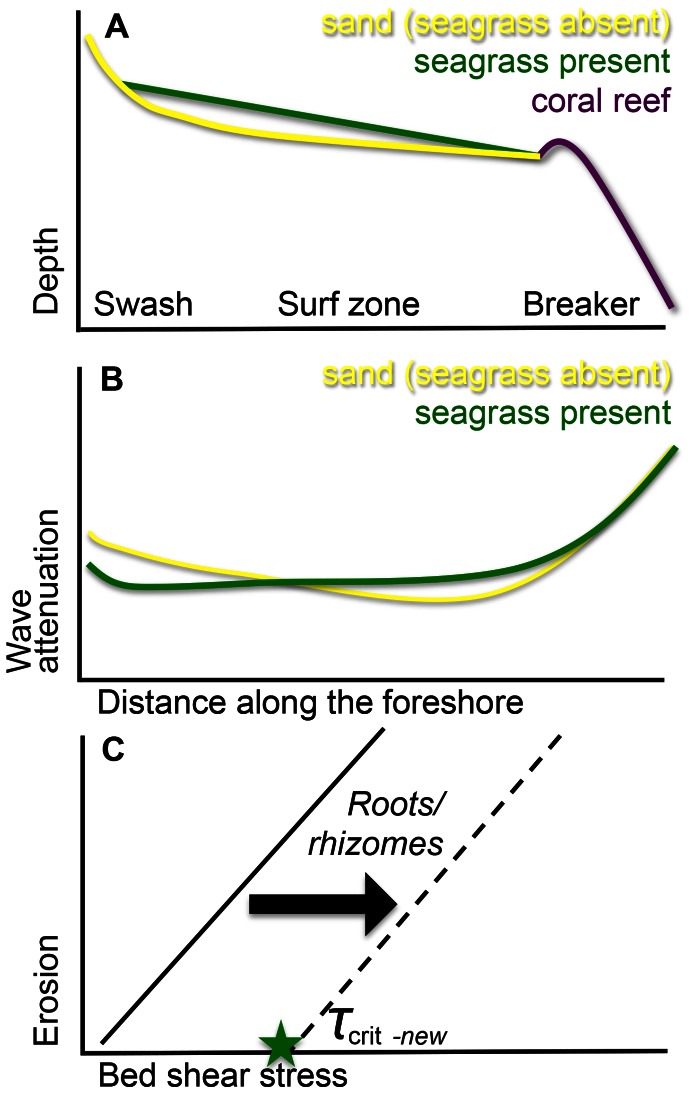
Conceptual model showing how erosion is decreased along a nearshore seagrass bed with a minimal canopy due to the combination of increased critical shear stress and resulting shallowness. Sediment erosion occurs when bed shear stress (force per unit area of the flow acting on the bed) exceeds a critical bed shear stress (τb > τcrit). (**A**) A typical depth gradient of a nearshore habitat where waves break above the coral reef, are then further reduced in the surf zone and “swash” onto the beach. Sediment stabilization by seagrass (green line) increases sediment bed levels compared to a situation with seagrass (yellow). (**B**) As a consequence of the reduction of the water depth by sediment stabilization of seagrass (green line), more wave energy is attenuated while travelling towards the shore compared to unvegetated areas (yellow), and less wave energy can reach the shore in the surf zone. This highlights the importance of seagrass with respect to coastal defense. (**C**) In the grazed seagrass meadow with short leaves and low-biomass, the low structural complexity of shoots in combination with the relative high root and rhizome biomass increases the critical bed shear stress that is needed for erosion (τcrit.).

The sediment stabilizing effect of grazed seagrass, which can even occur by low-biomass meadows, is expected to have important implications for both coastal protection and ecosystem functioning. With respect to coastal protection, by reducing sediment erodability, seagrass fields maintain a higher bed elevation that will help to attenuate waves. We have schematized these results in a conceptual diagram (Fig. 4). The sediment anchoring effect by short, grazed seagrass vegetation, which has most of its biomass in roots and rhizomes (Fig. 4c), increases the critical bed shear stress that is needed for bed erosion. We speculate that the presence of a dense mat of rhizomes and roots can have similar effects at the sediment-water interface as described for other biota that reduce erosion, such as biofilms of microphytobenthos [31]. Seagrass cover causes the sediment level to remain higher compared to eroded unvegetated gaps. In our study this was up to 13 cm, in others 18 cm [49](*Zostera marina*). Over longer time scales, this difference in erodability of the sediment is expected to seriously affect the form of the cross-shore height profile. The shallower profile of seagrass beds, compared to situations without seagrass, may imply that more wave energy is absorbed before waves reach the coastal strip (Fig. 4b), because dissipation of wave energy is a direct function of water depth [1]. As a result, it is expected that less wave energy can propagate over the nearshore towards the beach (Fig. 4b). It should however be detailed how this picture is influenced by wave breaking. In our study we observed wave breaking at the station closest to the shore, at least during part of the tidal cycle. Preferential zones of wave breaking could locally experience higher bottom shear stress and smaller-scale variations in the profile could arise, but this effect will decrease with the vegetation-induced stabilization of the sediment.

With respect to ecosystem functioning, armoring of the sediment can have profound implications for the subtidal seagrass community by the reduction of the amount of sediment that is resuspended. Biotic communities are known to suffer from sediment movement, due to processes such as direct smothering [50] or burial [51], and abrasion of tissues [52], [53]. The prevention of erosion by seagrass as a foundation species [54](Hughes *et* *al.* 2009) is further critical for burrowing fauna like shrimps that need stable sediment environments to reinforce their burrows [55]. Armoring by seagrasses may also indirectly protect the adjacent coral reef community that can suffer critically from sedimentation, by lowering sediment concentrations in the water column [4], [56].

More generally, our results show the stabilizing effects of macrophytes even when canopies are strongly reduced. This could also have important implications for other vegetated coastal ecosystems, such as salt marshes and dunes, as well. In our system, grazing by turtles was the main driver minimizing the canopy, but many other processes can have a similar effect, e.g. seasonal changes in aboveground biomass, shedding of leaves in autumn and winter or degradation due to high turbidity, epiphyte cover or eutrophication. We show, however, that these changes in canopy morphology do not automatically mean that seagrass beds have completely lost their coastal protection value. Although the relative value of seagrasses for coastal protection is strongly species dependent, with e.g. climax species (e.g. *Enhalus acoroides*) generally having a higher value than more ephemeral species (e.g. *Halodule univervis*) that can be highly variable in biomass and cover [57], even presence of low-canopy sea grass beds is significant. Therefore, when valuating seagrass habitats for coastal defense purposes, the idea of using aboveground biomass as a proxy for wave attenuation should be reconsidered. Such approach could greatly underestimate the coastal protection service of seagrass with canopies of low structural complexity. Seemingly insignificant low-biomass seagrass meadows that cover wide reef flats, may still offer significant coastal protection services, and should be valued as such. This ecosystem service is expected to become even more important in the near future, as storm frequencies are expected to increase and natural coastal protection structures like reefs are under on-going degradation [58].
